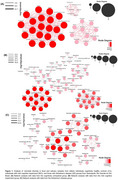# Alterations in Gut Microbiota Co‐Occurrence Networks in the Alzheimer’s Disease Continnum

**DOI:** 10.1002/alz70861_108953

**Published:** 2025-12-23

**Authors:** Eduarda Letícia Klafke Ebert, Amanda Muliterno Domingues Lourenço de Lima, Marco De Bastiani, Wyllians D Borelli, Isadora Crasnhak de Souza, Renan Antônio Barth, Joana Emilia Senger, Berenice Maria Werle, Laisa Zanella, Lilian Vivian Netson, Ariele Detogni, Neide Maria Bruscato, Joao Senger, Emilio Hideyuki Moriguchi, Rafaela Ramalho Guerra, João Vitor Cardoso Barboza, Andreza Francisco Martins, Eduardo R. Zimmer

**Affiliations:** ^1^ Universidade Federal do Rio Grande do Sul, Porto Alegre, RS Brazil; ^2^ Universidade Federal do Rio Grande do Sul, Porto Alegre, Rio Grande do Sul Brazil; ^3^ Universidade Federal de Ciências da Saúde de Porto Alegre, Porto Alegre, Rio Grande do Sul Brazil; ^4^ Instituto Moriguchi, Veranópolis, Rio Grande do Sul Brazil; ^5^ Hospital de Clínicas de Porto Alegre, Porto Alegre, Rio Grande do Sul Brazil; ^6^ Federal University of Rio Grande do Sul (UFRGS), Porto Alegre, RS Brazil

## Abstract

**Background:**

The gastrointestinal system hosts the intestinal microbiota—a diverse community of microorganisms, including bacteria, fungi, and viruses. Emerging evidence suggests that alterations in the intestinal microbiota may be linked to the development of Alzheimer's disease (AD). However, these evidences comes from studies in the Global North. This pilot study aims to explore whether gut bacterial communities are associated with the clinical stages of Alzheimer's disease (AD) in a small cohort in the south of Brazil.

**Method:**

This study was conducted with 12 elderly participants classified as cognitively unimpaired (CU), mild cognitive impairment (MCI), or Alzheimer's disease (AD). Fecal samples were sequenced using the Illumina MiSeq™ platform, targeting the V3–V4 regions of the 16S rRNA gene. The sequences were processed using the DADA2 pipeline, generating Amplicon Sequence Variants (ASVs) that were taxonomically classified with the SILVA database. To analyze microbial interactions, centered log‐ratio (CLR) transformed abundance data were used to construct correlation networks between genera across the cognitive groups.

**Result:**

A reduction in bacterial abundance and connectivity was observed across the stages of AD (Figure 1). Despite differences in abundance, certain bacterial groups were present at all stages, namely Lachnospiraceae, Bacteroidaceae, and Prevotella. Changes were observed in the genera *Prevotella_7* and *Bacteroides_pectinophilus_group*, which were abundant in CU individuals, but showed a gradual decrease in abundance in MCI and AD patients. *CAG_56* became reduced in MCI and absent in the AD group. Additionally, the interactions involving Roseburia and Megamonas were altered across the different stages of the disease.

**Conclusion:**

Our findings support the hypothesis that disruptions in the gut microbiota network are associated clinical stages of AD. The observed decline in microbial interactions and the persistence of certain bacterial groups across disease stages, suggests that those may play a role in the neurodegenerative process. Future studies involving a larger number of participants from this cohort are needed to explore potential interactions between microbiota‐gut‐brain and AD in this underrepresented population.